# Quantitative analysis of DNA with single-molecule sequencing

**DOI:** 10.1038/s41598-018-26875-7

**Published:** 2018-06-04

**Authors:** Takahito Ohshiro, Makusu Tsutsui, Kazumichi Yokota, Masateru Taniguchi

**Affiliations:** The Institute of Scientific and Industrial Research, Osaka University, 8-1 Mihogaoka, Ibaraki, Osaka, 567-0047 Japan

## Abstract

Cancer can be diagnosed by identifying DNA and microRNA base sequences that have the same base length yet differ in a few base sequences, if the abundance ratios of these slightly deviant base sequences can be determined. However, such quantitative analyses cannot be performed using the current DNA sequencers. Here we determine entire base sequences of four types of DNA corresponding to the *let*-7 microRNA, which is a 22-base cancer marker. We record the single-molecule conductances of the base molecules using current-tunneling measurements. In addition, we count the numbers of molecules in a solution to determine the abundance ratios of two DNA strands that differ by a single base sequence.

## Introduction

Genetic information has the potential to drive personalized medical treatments in the near future. Because the information necessary for personalized medicine is unique to an individual, researchers often assume that an individual’s sequenced genome will be sufficient for medical purposes. However, diagnostic processes require information about the gene expression along with sequences of DNA and microRNA^1–10^. For example, in cancer marker microRNA strands, eight types of microRNAs are suppressed only when a human has cancer^4–8^. Even in a healthy patient, eight types of microRNA are expressed; however, the onset of cancer changes the abundance ratio of these eight microRNA sequences. Hence, genomic diagnosis requires a method that simultaneously measures the amounts of DNA or microRNA with a specific base sequence.

The real-time polymerase chain reaction (RT-PCR) method is used widely for determining the amount of DNA containing a known base sequence^11–13^. In contrast, DNA sequencers have evolved in recent years. First- and second-generation DNA sequencers amplify DNA using PCR and detect dye-modified base molecules using light as the probe^10,14–17^. Next-generation DNA sequencers detect base molecules using pH and ionic current as probes, which eliminates the need for PCR amplification and dye modification^15,16,18^. The nanopore sequencer using ionic current as a probe is expected to be able to determine the amount of DNA in principle, but the results of quantitative analysis have not been reported so far. Existing DNA sequencers can determine the base sequence of DNA that includes a single type of base sequence but cannot determine the amount of DNA present. In addition, because the DNA microarray method can examine the amount of DNA and microRNA molecules with two or more known base sequences, it is widely used for disease research that relies upon DNA or microRNA^19–22^.

However, the current methods cannot determine the base sequence and quantities of DNA and microRNA simultaneously, i.e., they cannot be used to perform quantitative analysis. Furthermore, personalized medicine applications require a method that does not involve chemical modification using expensive reagents and protracted PCR amplification, to allow high throughput at low cost.

Determination of DNA base sequences and partial amino acid sequences of peptides is possible with the measurements of the tunneling current flowing through the base molecules that are set between electrodes^23^. This method of reading the difference in the electronic state of a single molecule through tunneling current does not require chemical modification of DNA or amplification by PCR because this method measures single molecules, in principle. Furthermore, because the method measures individual DNA molecules, we expect that two or more base sequences can be determined by measuring a solution of DNA molecules with two or more types of base sequences. In addition, because this method can count the number of DNA molecules that contain a specific base sequence, we expect that the quantitative analysis can detect the base sequences and counts their instances.

## Results and Discussion

### Single-molecule sequencing method

The tunneling currents flowing through single molecule were measured by gold nanogap electrodes using a mechanically controllable break-junction^24–26^. The two electrodes were separated by 0.75 nm with each other, equivalent to the size of a DNA base molecule. This distance was maintained by a feedback control while monitoring the tunneling current (*I*). The electric conductance (*I*/*V*)–time profiles of the DNA solutions were measured under a bias voltage of *V* = 0.1 V. MicroRNAs are 18–25 bases long RNA that control gene expression^4–8^. The *let*-7 microRNA is an especially important cancer marker that infers the diagnosis, stage, progression, and prognosis of human cancer. As microRNAs degrade in the atmosphere, we investigated the DNAs corresponding to four kinds of *let*-7.

**Figure 1:**
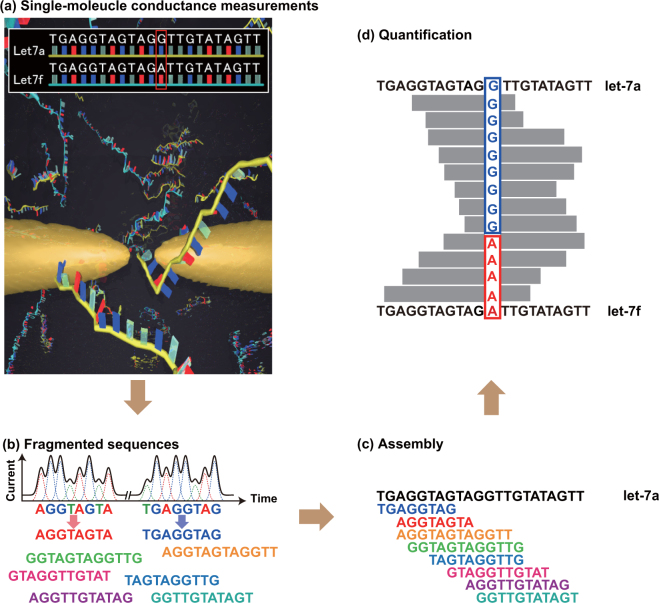
Process of quantitative DNA analysis by quantum sequencing, adapted to the DNAs corresponding to the microRNA cancer marker *let*-*7*. (**a**) Measure the current–time profiles of aqueous solutions containing two kinds of DNA. (**b**) Determine the fragmented base sequences. (**c**) Assemble the fragmented sequences to obtain the whole sequence. (**d**) Determine the abundance ratios of the two DNAs using base molecules specific to the DNAs as a marker.

To identify the DNA base sequences corresponding to *let-*7 and their abundance ratios in solution, we first measured the tunneling current–time profiles for one or two types of solubilized DNAs, and determined different fragmented sequences by our base-calling method, which is similar to the Phred method widely used in genome sequencing (Fig. 1b)^27,28^. Subsequently, the whole base sequence was determined by an assembly method that glues the consensus fragments to reconstruct the original sequence (Fig. 1c). Selecting the marker as a base molecule specific to a certain DNA, and presuming that a fragmented sequence containing the marker corresponds to a DNA having that marker, we can determine the abundance ratios of two DNAs with different markers by counting the fragmented sequences in a solution of the mixed DNAs (Fig. 1d).

### Determination of partial sequences of DNA

**Figure 2:**
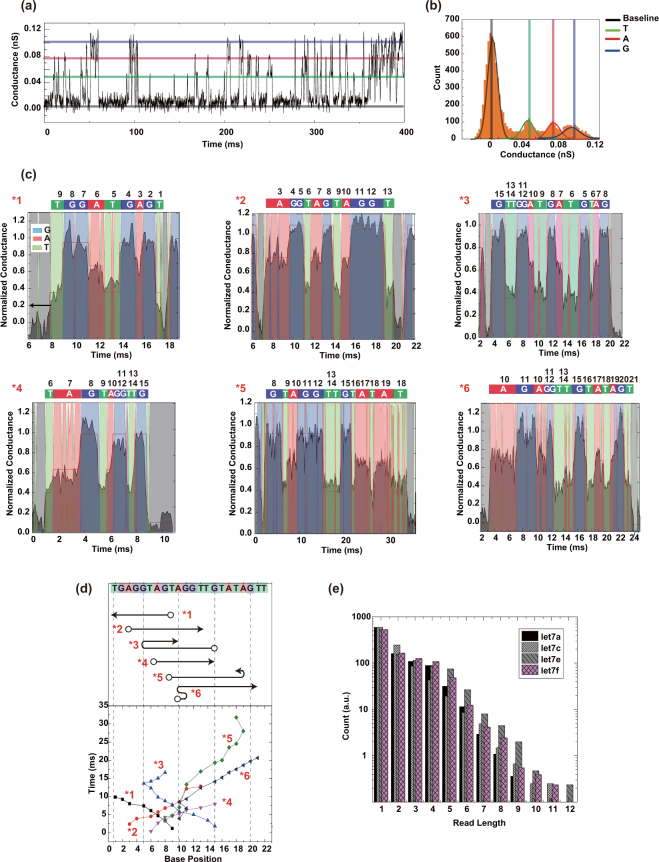
Determination of fragmented sequences of DNAs consisting of 22 base molecules. (**a**) Typical single-molecule conductance–time profile of aqueous solutions containing DNAs corresponding to *let*-*7a* and *let*-*7f*. The blue, red, and green lines indicate the peak conductances of guanine, adenine, and thymine, respectively. (**b**) Count histogram of measured conductances in the profile of (**a**). The curves are fitted by the Gaussian distribution. Gray indicates the baseline; the other color codes are described in (**a**). (**c**) Fragmented sequences *1 to *6 obtained from the electrical conductance–time profiles of DNA corresponding to *let*-*7a*. Fragmented sequence *1 represents TGAGTAGGT. The vertical axis is the conductance normalized by the conductance of guanine. (**d**) Transition-point analysis of fragmented sequences *1 to *6. The symbol ○ indicates a read-start base. Starting from the 9th T in the fragmented sequence of *1, the base sequence is sequentially read out in the direction of the arrow. (**e**) Read-length histograms of fragmented DNA sequences corresponding to the four variants of *let*-*7*.

The typical electrical conductance–time profile of DNA corresponding to *let*-7*a* (TGAGGTAGTAGGTTGTATAGTT) exhibits spike-like signals at three intensity levels (Figs 2a and S1). Figure 2b presents a count histogram of the conductance data collected from 0 to 400 ms. The occurrence frequency peaked at 45 pS, 77 pS, and 102 pS. The single-molecule conductance of each base was corrected using the baseline defined as the minimal signal level in the conductance profile. Comparing the results with reported single-molecule conductances of base molecules, the lowest, intermediate, and highest conductance peaks were attributed to thymine, adenine, and guanine, respectively^24,26^.

After automatically extracting the signals (continuously changing electrical conductances at ≥6*σ* above the base current noises) from the electrical conductance–time profiles, the fragmented sequences were stochastically determined from the single-molecule conductance histograms, which correspond to the probability density functions of the conductance of each base molecule. When assigning a signal to a base molecule, we assume that the base molecule translocates only between its adjacent base molecules. Specifically, we divided the electrical conductance–time profile into 0.5-ms intervals, and determined the most probable molecular species in each interval by integrating the probability density functions within the interval. The selected interval (0.5 ms) was the experimentally determined minimum retention time of one base molecule in the vicinity of the electrodes. As a part of the DNA molecules stochastically passes through the nanogap electrode by Brownian motion, many fragmented sequences were obtained randomly. Typical fragmented sequences are shown in Fig. 2c. The obtained fragmented sequence was TGGATGAGT (labeled as *1 in Fig. 2c), but was assigned as TGATGGAGT after referring to the base sequence of the measured DNA. This indicates that the base sequence was read out in one direction. Similarly, the base sequences of AGGTAGTAGGT and TAGTAGGTTG (*2 and *4 respectively in Fig. 2c) were read out in one direction. Interestingly, the reading direction of GTTGGATGATGTAG (*3 in Fig. 2c) reverses from the G at base position 5 to the G before the terminal TAG, causing a duplicated readout. The time dependency of the read base molecules clarifies the transition point in the reading direction. Transitions in the readout direction are also observed in the 5th (*5) and 6th (*6) partial sequences in Fig. 2c. Such transitions suggest that the DNA flow dynamics near the nanogap electrode are influenced by local electric fields of the nanogap electrode (which generate electrophoresis effects), Brownian motion, and DNA–electrode interactions^29,30^. Analyzed by their transition points, the fragmented base sequences have very similar slopes on a time versus base-position plot (Fig. 2d). This suggests that when the base sequence is read out, the DNA molecule passes through the nanogap electrode at an approximately uniform velocity of 1.5 bases/ms. The readout of the fragmented DNA sequences corresponding to *let*-7*a*, *let*-7*c*, *let*-*7e*, *let*-*7f* admitted up to 12 base molecules (Fig. 2e). The read length was independent of the base sequence, and the number of readouts decreased exponentially with read length.

### Determination of whole sequences of DNA

**Figure 3:**
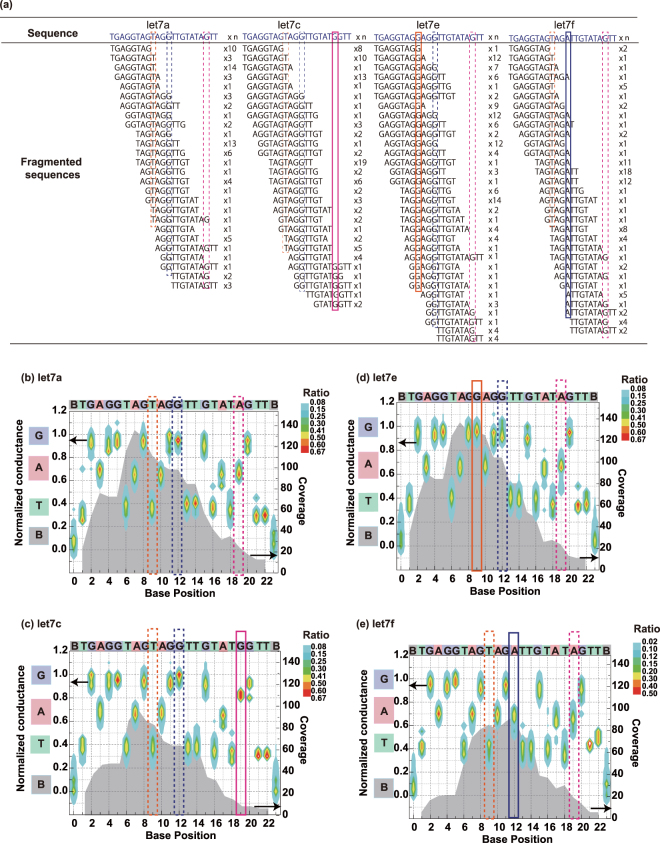
Determination of whole base sequences of DNAs corresponding to four kinds of *let*-*7*. (**a**) Fragmented sequences and the numbers of sequences with seven bases or more, used for determining the whole base sequences. The sequences of the four DNAs differ only by a single base. Base molecules that differ from *let*-*7a* at each position are enclosed in pink, orange and blue rectangles. Heat maps of DNA corresponding to (**b**) *let*-*7a*, (**c**) *let*-*7c*, (**d**) *let*-*7e*, and (**e**) *let*-*7f*, constructed from fragmented sequences of five or more bases. The left and right axes are the conductance normalized by the conductance of G and the coverage of each base molecule, respectively. Gray areas indicate the coverages.

The whole sequences of the four DNAs were determined by assembling the fragmented sequences of five or more bases. The assembly process automatically adjusted the duplicated readout parts to the correct linear sequences. Figure 3a shows typical fragmented sequences of seven or more bases. Many of the fragmented sequences comprised less than four bases. Such short fragments were not used for assembly because they are difficult to assign to whole sequences. Panels b–e of Fig. 3 display heat maps of each base molecule prepared from the fragmented sequences used in the assembly. The heat maps verily the correct assemblages of the four kinds of DNA. The approximate conductances of adenine and thymine (normalized by the single-molecule conductance of guanine) in all four DNAs were 0.7 and 0.4, respectively. The coverages (number of readout times) of each base molecule were high near the centers of the four type of DNA molecules. This explains why the DNA entered the nanogap electrode from the 3′ or 5′ end directions with equal probability. The base coverages were higher near the centers than at both ends because fragmented sequences with fewer than five bases were discarded. The sequencing error in typical DNA sequencers decreases with increasing coverage (See Supporting Information). The minimum coverages of *let-7a*, *let*-*7c*, *let*-*7e*, and *let*-*7f* were 7, 6, 11, and 6, respectively. As the assignment accuracy of this analysis was 75% or higher, these coverages imply sequencing errors of 4.5% or less over the whole base sequences, sufficient for correctly sequencing the four DNAs. In particular, the coverages of the 19th, 9th, and 12th base positions of *let*-*7c*, *let*-*7e*, and *let*-*7f* respectively, which differ by one base from *let*-*7a*, are above 10. Therefore, the four DNAs can be distinguished with low errors.

### Quantitative analysis

**Figure 4:**
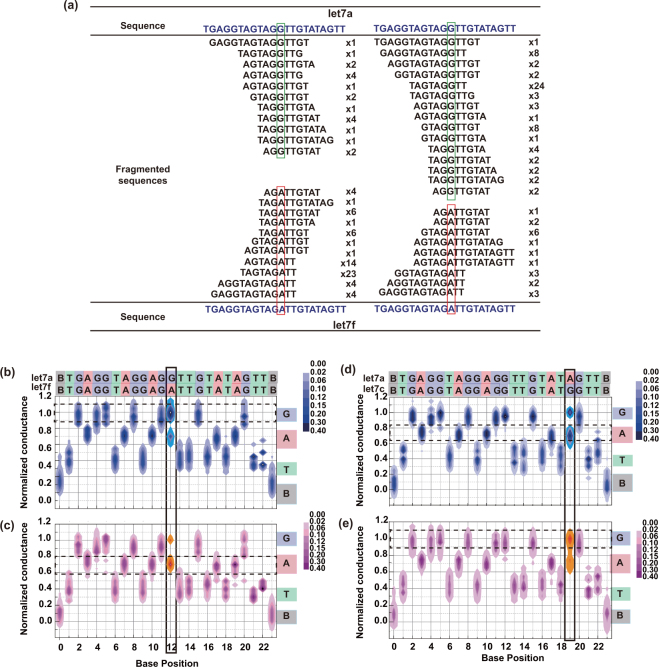
Quantitative analysis of solutions containing two DNAs corresponding to *let*-*7*. (**a**) Fragmented sequences and the numbers of sequences with eight bases or more obtained from a mixed solution of DNA corresponding to *let*-*7a* and *let*-*7f*. Heat maps generated at *let*-*7a* and *let*-*7f* molar mixing ratios of (**b**) 3: 1 and (**c**) 1: 3. Heat maps generated at *let*-*7a* and *let*-*7c* molar mixing ratios of (**d**) 3: 1 and (**e**) 1: 3. The marker bases of each DNA, used in the quantitative analysis, are enclosed in black rectangles. Fragmented sequences of five or more bases were used for profiling.

The abundance ratios in a mixed solution of two DNA types were determined from the heat maps and by whole-base sequencing. The electrical conductance–time profiles of the mixed-DNA solution were measured, and the fragmented sequences were determined as described for single molecule DNA. The *let*-7*f* sequence is the *let*-*7a* sequence with one G replaced by A (Fig. 4a). Both molecules are markers that discriminate between two *let* genes, and their mixing ratio in solution is expected to equal the total number ratio of the fragmented sequences containing G and A. The fragmented sequences containing the markers and at least five bases of *let*-7*a* and *let*-7*f* were assembled into the total base sequences of the respective DNAs. (panels b and c of Fig. 4). The normalized conductance of the 12th base position in the heat maps was 1.00 and 0.72 for G and A, respectively, consistent with the heat maps of the single DNAs. The abundance ratios of *let*-*7a* and *let*-*7f* were quantified as 2.6: 1.0 and 1.0: 2.4 at charged molar mixing ratios of 3:1 and 1:3, respectively. Similarly, the abundance ratios of *let*-*7a* and *let*-*7c* were 2.8: 1.0 and 1.0: 2.4 at charged molar mixing ratios of 3:1 and 1:3, respectively. The whole base sequences of both DNAs were determined (Fig. 4d and e). Recall that in *let*-7*c*, one A of *let*-*7a* is replaced by G. According to the heat maps, the normalized conductance of the 19th base position was 1.00 for G and 0.73 for A, consistent with the heat maps of a mixed solution of *let*-*7a* and *let*-*7f*.

### Conclusions

Sequencing DNA that includes two types of base sequences can be realized by measuring single-molecule conductance and determining the abundance ratio in solution by counting the molecular number. In the sequencing process, we found that the single-molecule sequencing method can also be used for investigating the fluid dynamics of a single DNA molecule in solution. This suggests that the process can also be applied to the analysis of microRNA and RNA molecules that include four base molecules and peptides that include 20 kinds of amino acids because the single-molecule sequencing method detects differences in the electronic states of molecules in terms of single-molecule conductances. Furthermore, because the proposed quantitative analysis method can detect chemically modified base molecules and amino acids, the method is a significant step toward realizing personalized genomic diagnosis of cancer and other diseases.

## Methods

### Preparation of nucleotide solutions

All oligonucleotide samples were purchased from Hokkaido System Science Co., Ltd. and Sigma Aldrich Co. Ltd. and purified by high-performance liquid chromatography. The sample molecules were used without further purification. The nucleotide sample was soluted in MilliQ water and the sample solution was 1 mM phosphate buffer. The concentration of each nucleotide (*let-7a*, *let-7c*, *let-7e*, and *let-7f*) was 1 μM. In the mixed sample solutions (*let-7a*/*let-7f* and *let-7a*/*let-7c*), the nucleotides were present at molecular ratios of 1:3 or 3:1, and the total nucleotide concentration was adjusted to 1 μM. The nucleotide concentrations were estimated by measuring the OD_260_ (E-spec).

### Electrical measurements

The electrical properties of the nucleotides and oligonucleotides were measured at the optimal gap distance of the nanogap electrodes. Experimentally, the optimal gap was determined as 0.75 nm, comparable to the size of the nucleotide molecules. A lithographically fabricated gold nanowire on a thin polyimide-coated phosphorous bronze substrate was broken by mechanically bending the substrate at 300 K in air. After reconnecting the gold nanowire, a constant DC bias voltage of 0.1 V was applied, and the nanowire substrate was gradually bent using a piezoactuator. Throughout the junction breaking process, the junction conductance (*G*) was monitored by a picoammeter (Keithley 6487). A series of conductance jumps of the order of *G*_0_ = 2*e*^2^/*h* (where *e* and *h* are the electron mass and Planck’s constant, respectively) was observed, and the final conductance was 1 *G*_0_. Several seconds after reaching the 1 *G*_0_ state, a gold–gold atomic contact was naturally ruptured in the nanowire, creating a pair of electrodes. The gap size was determined as 0.5 nm. By controlling the piezovoltage, the electrode gap distance was increased to 0.8 nm for the sample nucleotide measurements.

### Signal detection procedure

Signal detection was carried out according to the following procedure: First, the value of the current in the region where the molecules do not pass between the nanoelectrodes was considered as the base current. This base current value was defined as a moving average value of 2000 data points. Next, it was assumed that the current value follows the normal distribution when the molecule to be measured does not pass between nanoelectrodes. Under this assumption, it was presumed that the data region beyond the base current is assumed as a signal. When the value obtained by subtracting the base current value from the measured current value (raw data) exceeded six times the standard deviation of the normal distribution, it was considered that the amplitude of the signal increased. Thereafter, when the value obtained by subtracting the base current value from measured current value was less than 1 times the standard deviation of the normal distribution, it was considered that the amplitude of the signal has decreased. The current–time domains satisfying both the rise and fall were detected as signals. The standard deviation values used here were determined for each one-second region. The values for the standard deviation in the one-second region were defined as the most frequent values of the set of standard deviation values in each 20-data region obtained by dividing the data (10,000) of the one-second regions into 500 parts.

### Base assignment procedure

The signals of each sample nucleotide were measured on 20 mechanically controllable break-junction (MCBJ) chips. On average, 1000 signals were obtained on each chip, and at least 10,000 signals were collected for the base-calling and signal-assembly analyses. Typical conductance–time profiles are shown in Figs 2a and S1. To clarify the base-dependent tunnel current, we must first reduce the conductance fluctuations (which include the electrical measurement noise). At a data acquisition rate of 10 kHz, the minimum discernible transition time (determined from the transition-time profile data) was 0.5 ms. Therefore, we set 0.5 ms as the minimum retention time around the sensing electrode, and ignored the abrupt (<0.5 ms) conductance changes. Using the smoothed *I–t* profiles, we constructed the current histograms. The conductance histograms correspond to the probability density functions of the base molecules. The lowest and highest conductance peaks were assigned to the baseline and guanine, respectively. Comparing the peak conductances with previously reported mononucleic acid conductances, we assigned the other conductance peaks in the histograms to thymine, adenine and guanine species in the signals. Using the calculated probabilities of each base-species and the baseline, we determined the presented types of base molecules.

## Electronic supplementary material

Supplementary Information
