# Supplementary material for: Quantitative analysis of DNA with single-molecule sequencing
Source: Sci Rep. 2018 Jun 4;8:8517. doi: 10.1038/s41598-018-26875-7 (PMC5986817; doi:10.1038/s41598-018-26875-7)
Supplement: Supplementary file 1 — Supplementary Information [file 41598_2018_26875_MOESM1_ESM.pdf]

Supplementary Information for

Quantitative analysis of DNA with single-molecule sequencing

Takahito Ohshiro, MakusuTsutsui, Kazumichi Yokota, and Masateru Taniguchi

The Supplementary Information includes:

1. Supplementary Text

2. Supplementary Figure (Fig. S1)

3. Supplementary References

1. Continuous-base identification.

Continuous single-base sequences such as GG were not discriminated in the single-base signals, *i.e.*, from the conductance differences G, because the conductances of both bases are comparable in the time profiles. However, when the flow rate of the unidirectional read region is relatively steady, such sequences can be identified by their longer retention time in the profile. To compare the G and GG retention times, we define the relative retention time t_x of each base as a retention-time (t_G or t_{GG}) per average flow speed t_0, where t_0 is the slope of the linearly fitted data in the base-position (#) versus time (t) profiles. For example, in *let-7e* (TGAGGTAGGAGGTTGTATAGTT), the relative retention times of singlet G at #2 and #15, (*i.e.,* $t_{\#2(G)}$ and $t_{\#15(G)}$) were 1.70 and 2.04, respectively. On the other hand, the relative retention times of double GG at #4–5 and #8–9 (*i.e.,* $t_{\#4,5(GG)}$ and $t_{\#8,9(GG)}$) were 2.92 and 2.66, respectively. As the GG retention times were noticeably longer than the G retention times, the discrimination between G and GG is statistically possible. However, as t_G was not simply half of t_{GG}, further quantitative discrimination of contiguous bases such as G, GG, GGG, and GGGG requires a steady flow analysis at each base-transition behavior.

2. Accuracy of resequencing based on contig assemblies.

The accuracy of a determined sequence (*Pr*) is determined by the depth of its coverage (*d*), defined as the average number of reads representing a given nucleotide in the reconstructed sequence, and the assigned accuracy (*p*) of each base[S1]. The error probability is then calculated as follows:

$$Pr = 1 - exp\left\{-\left(\frac{p}{1-p}\right)^d\right\},$$

According to this equation, increasing the depth *d* increases the accuracy of the determined sequences. When assembling the fragmented sequences shown in Fig. 3a, we used only the fragments exhibiting more than seven right-read signals ($d > 7$) with accuracies exceeding 75% ($P > 0.75$). The error probability of the assembly (Fig. 3a) was below 0.045, sufficient for identifying the 22 base-pair sequence *let-7a*, *c*, *e*, and *f*.

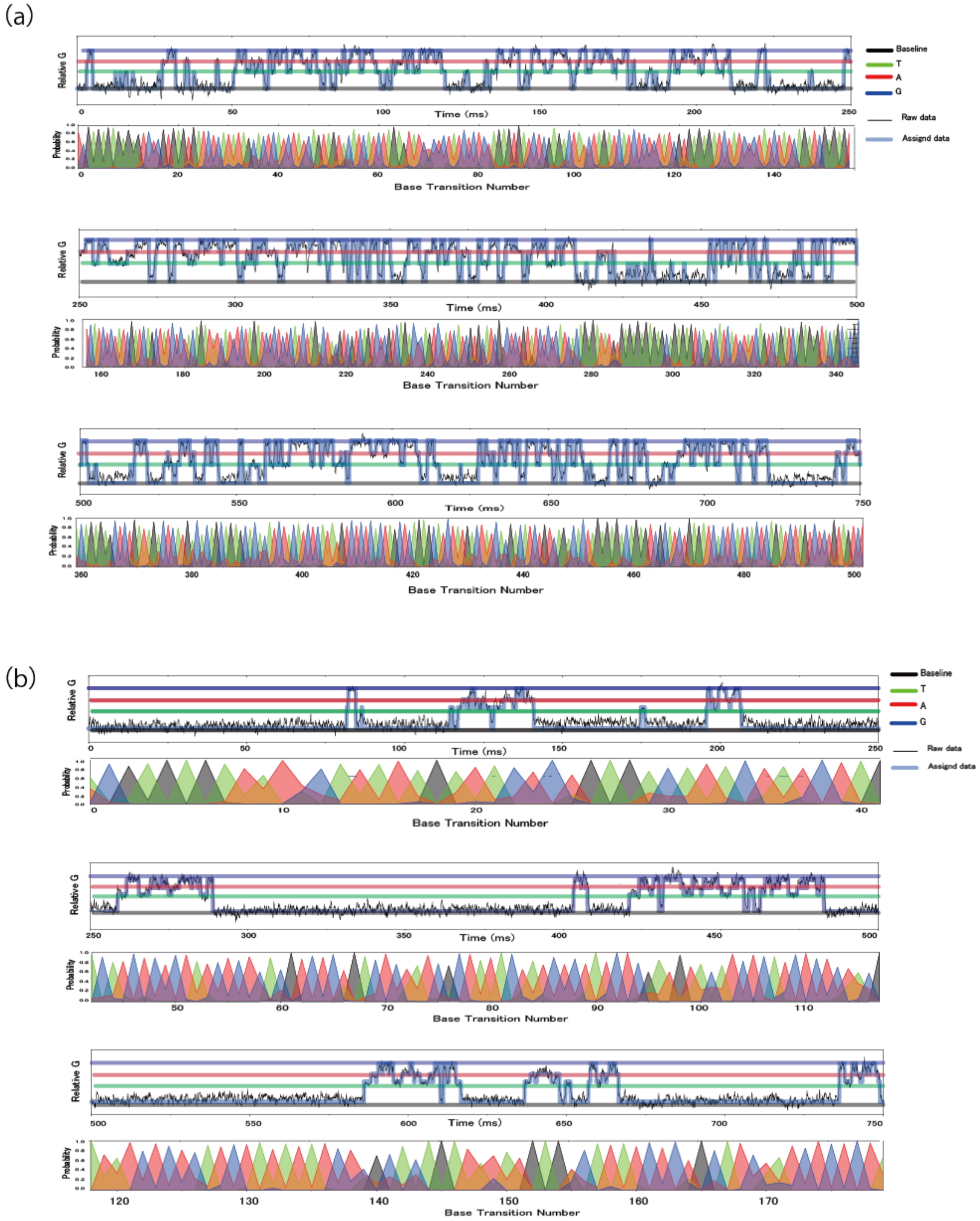

Figure S1 Conductance–time (*G*–*t*) profiles of (a) *let-7c* and (b) *let-7e*. The three conductance levels represent guanine (blue), adenine (red), and thymine

(green). Gray lines show the baselines. The raw data (black) in the *G*–*t* profiles are superposed with the assigned conductance data (blue). The base-probability profiles are revealed in the raw data. The most probable base species in each successive time region was assigned to that time region.

Supplementary Reference

S1. Churchill, G. A. & Waterman, M. S. The accuracy of DNA sequencers: Estimating sequences quality. *Genomics*.**14**, 89-98, (1992).
